# 持续负压引流在口咽部来源急性前纵隔感染中的治疗经验

**DOI:** 10.3779/j.issn.1009-3419.2018.04.23

**Published:** 2018-04-20

**Authors:** 安平 陈, 刚 徐, 剑 李, 永祥 宋, 庆勇 蔡

**Affiliations:** 563000 遵义，遵义医学院附属医院胸外科 Department of Thoracic Surgery, Affiliated Hospital of Zunyi Medical College, Zunyi 563000, China

**Keywords:** 纵隔感染, 负压引流, 纵隔脓肿, Mediastinal infection, Negative pressure drainage, Mediastinal abscess

## Abstract

**背景与目的:**

纵隔感染是累及纵隔结缔组织的严重感染，并发症较多且死亡率较高；治疗上应用广谱抗生素及营养支持外，早期充分引流为成功救治的关键；在引流方式上，我们应用持续负压引流技术治疗严重口咽部来源的急性前纵隔感染取得较好效果，在此予以总结分享。

**方法:**

2017年1月-12月我院共收治17例来源于口咽部急性纵隔感染，已形成纵隔脓肿，手术均采用胸骨后对口负压引流方式，即经胸骨上窝及剑突下切口游离胸骨后前纵隔间隙，使之贯通并放置引流装置，缝合封闭创口，持续负压引流，负压采用3 cm-5 cm水柱。

**结果:**

17例患者中，14例患者经持续负压引流引流液清亮感染消退，然后拔除引流管；2例患者感染破溃入右侧胸腔，行闭式引流术导致负压消失，放弃负压引流改用常规引流，引流管液体清亮后拔除引流管；1例患者已经形成纵膈脓肿切开引流时间较晚，并发感染性休克、脓毒血症，最终导致多器官功能衰竭死亡。

**结论:**

严重急性纵隔感染传统的治疗方法是胸骨切开引流，患者创伤大、心理难以承受，且医生工作负担较重；持续负压引流术减轻患者痛苦、能够充分引流纵隔积液，并且避免了敷料反复渗出而需要的换药，对治疗严重急性前纵隔感染是一种有效的方法。但该方法对中、后纵隔引流存在局限性，有待进一步优化。

## Abstract (en)

**Background and objective:**

Mediastinal infection is a serious infection of mediastinal connective tissue, with more complications and higher mortality. Application of broad-spectrum antibiotics and nutritional support, early sufficient drainage is the key to successful treatment. In the mode of drainage, this paper discusses the application of continuous negative pressure drainage technique to treat acute anterior mediastinal infection of severe mouth pharynx source, and the good results are summarized and shared.

**Methods:**

In January to December in 2017, a total of 17 cases treated acute mediastinal infection is derived from the throat, has formed a mediastinal abscess, surgery adopts retrosternal counterpart negative pressure drainage way, namely the sternum nest and free sternum xiphoid process under the incision on the first mediastinal clearance, make breakthrough and placed drainage device, suture closed wound, continuous negative pressure drainage, negative pressure using 3 cm-5 cm water column.

**Results:**

Among the 17 patients, 14 patients were relieved by continuous negative pressure drainage, and then the drainage tube was removed. In 2 cases, the infection broke into the right thoracic cavity, and the closed drainage caused the negative pressure to disappear, and the negative pressure drainage was replaced by the conventional drainage, and the drainage tube was removed after the drainage tube was clear. One patient had formed a mediastinal abscess incision drainage time later, complicated with septic shock and sepsis, resulting in the death of multiple organ failure.

**Conclusion:**

The traditional treatment of severe acute mediastinal infection is sternal incision and drainage. Continuous negative pressure drainage adequate drainage of mediastinal can relieve patients' pain, effusion, and avoid the dressing out repeatedly. It is an effective method. However, there are limitations in this method, which need to be further optimized.

纵隔感染是一种累及纵隔结缔组织的严重感染^[1]^。在积极全身广谱抗生素应用并根据药敏结果选用抗生素以及全身支持治疗下，患者的死亡率仍高达25%-50%^[2]^；在纵隔感染中充分引流起到至关重要的作用。我科2017年1月-12月共收治前纵隔感染患者17例，纵隔感染来源于口咽部感染，经颈前间隙蔓延至胸骨后间隙，我们采用胸骨后持续负压引流方式进行治疗，取得较好疗效，现报道如下。

## 1 材料和方法

### 1.1 一般资料

总结分析我科2017年1月-12月收治17例纵隔感染患者，其中男性12例，女性5例，年龄17岁-75岁，平均46.8岁；住院时间2天-38天，平均21.6天；感染诱因为继发口咽部感染14例，为入院前1周有拔牙5例、牙龈感染5例、咽喉肿痛4例；3例无明确感染源；主要症状：发热、牙龈肿胀、颈部肿胀、疼痛、咽痛、吞咽疼痛，部分牙龈肿胀者出现张口困难、进食困难等症状；17例患者中5例患有糖尿病，所有人均伴有不同程度的低蛋白血症。

### 1.2 治疗过程

所有患者均在全身麻醉气管插管下于胸骨上窝2横指处取横切口长约2 cm，分离颈前间隙至气管前间隙，进入脓腔后可见脓性液体流出，并闻及恶臭；然后行剑突下纵切口长约2 cm，经剑突后方分离胸骨后间隙，使胸骨后组织间隙上下贯通，将脓液留取标本行细菌培养，脓腔反复用生理盐水冲洗后，分别放置一次性硅胶负压引流管（陕西省京典生物科技有限公司，19号引流管，十字凹槽型，凹槽长度约40 cm，引流球规格：400 mL），缝合皮肤封闭创口，并用缝线固定引流管，引流管另一端与伤口负压引流瓶相连接，持续负压引流；同时给予广谱抗菌素及控制脓毒血症、纠正低蛋白血症、维持电解质平衡、静脉补充脂肪乳支持等治疗，并积极治疗糖尿病等基础疾病。需密切记录引流管24 h引流量，隔日行引流液培养；术后第1天常规复查床旁胸片、血常规、血生化、降钙素原及痰细菌培养；发热38.5 ℃常规行血细菌培养；并于术后3天-5天复查胸部计算机断层扫描（computed tomography, CT）了解引流情况。

## 2 结果

本组17例患者中，14例患者经持续负压引流至引流液清亮、体温和血象正常后，剪断引流管变开放引流，然后逐渐退除引流管；2例患者纵隔感染破溃进入右侧胸腔，行胸腔闭式引流术，负压消失改用无负压引流方法治疗；1例患者纵隔脓肿形成1周后入院切开引流，已经出现感染性休克、脓毒血症，最终多器官功能衰竭死亡。所有患者均行负压封闭引流术后，12例带气管插管呼吸机辅助呼吸返回病房，术后第2天均拔除气管插管，1例老年患者再次插管并行气管切开，1周后停用呼吸机；使用呼吸机辅助期间需行胃肠减压，完全肠外营养支持。待患者停用呼吸机、消化道排气后拔除胃管，行肠内营养支持；因所有患者术后均有不同程度的低蛋白血症，所以均予以肠内高营养支持同时，予以静脉脂肪乳、氨基酸等治疗；术后需密切观察引流情况，该组患者可见引流液为浑浊脓液并伴有恶臭。患者在15天-20天时引流液清亮后再次复查胸部CT及去除负压装置，剪断引流管开放引流，逐步退出引流管，待引流管全部退出，切口皮肤完全愈合。13例患者脓液细菌培养结果为星座链球菌：3例为肺炎克雷伯菌肺炎亚种，1例无细菌生长；血培养均为阴性。本组1例患者术后出现持续高热、感染性休克、双侧胸腔积液等并发症，患者家属拒绝进一步手术治疗签字出院。

典型病例一：

患者xx，75岁，女，2017.08.16因“胸骨上部疼痛6天余”入院。查体：T 36.5 ℃，P 90/分，R 20次/分，BP 150 mmHg/70 mmHg，急性病容，神志清晰，胸骨上段触痛明显，皮肤潮红，局部皮温高，双下肺闻及少许湿罗音；入院胸部CT（图 1A1）：胸骨柄周围、前纵隔见软组织增厚，可见液性暗区及软组织积气，胸骨体骨折，考虑感染性病变。B超：胸骨柄右侧肌同隙混合回声团，考虑脓肿可能性大。血常规：WBC：25.25×10^9^/L，NEUT%：82%，RBC：3.03×10^12^/L，HGB：90.0 g/L；PLT：456×10^9^/L。治疗经过：立即行上纵膈脓肿病灶清除及持续负压引流术，术后子常规抗感染、化痰、营养支持等治疗，引流液分泌物行细菌培养提示星座链球菌，根据微生物结果应用对应抗生素抗感染，经治疗后10天复查胸部CT（图 1A2）示纵隔内软组织周围肿胀较前消退，引流管在位，胸骨内感染较前好转；继续治疗后于2017.09.15完全拔除引流管出院。

**1:**
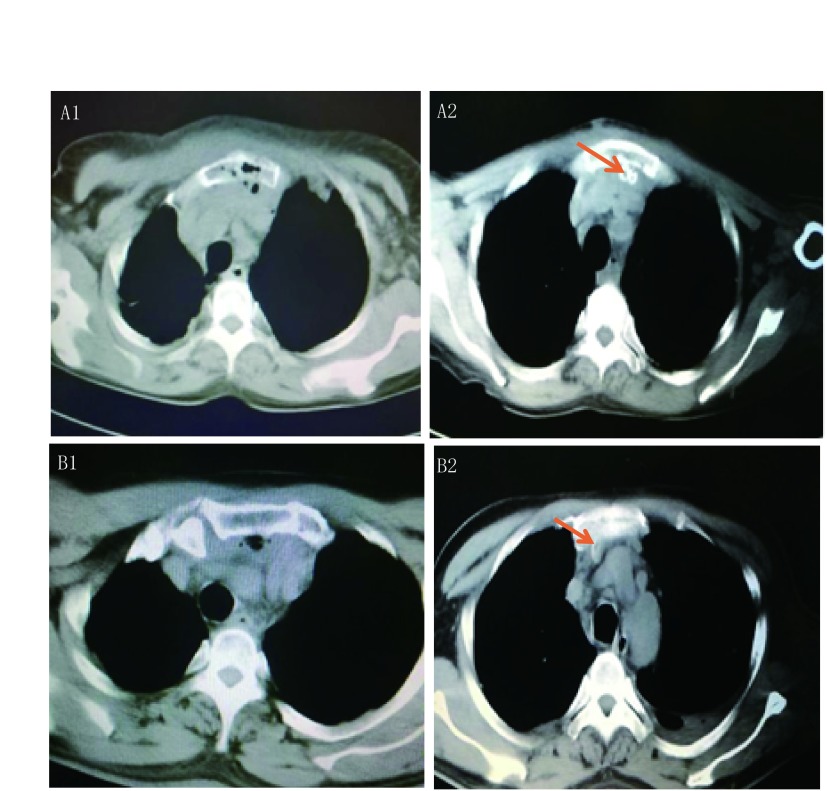
典型病例图片。A1：见胸骨柄及前纵隔周围软组织肿胀，见液性暗区及积气，胸骨骨折及胸骨内积液积气；A2：胸骨后方前纵隔区见引流管，周围软组织肿胀及胸骨内积气前明显好转；B1：胸骨后间隙见软组织肿胀及积气，考虑感染脓肿形成；B2：胸骨后方软组织肿胀消退，可见引流管。 Typical case pictures. A1: The soft tissue swelling around the sternum and anterior mediastinum is seen in the fluid dark area and the accumulation of gas, the sternum fracture and the accumulation of fluid in the sternum; A2: The anterior mediastinal region of the sternum was seen in the drainage tube, and the surrounding soft tissue was swollen and the sternum was significantly improved; B1: The posterior space of the sternum, soft tissue swelling and gas accumulation were observed, and the formation of infected abscess was considered; B2: The swelling of the soft tissue behind the sternum was subsided and the drainage tube was visible.

典型病例二：

患者XX，49岁，男，2017.11.16因“咽痛3天，左颈部肿痛1天”入院。入院查体：T 36.9 ℃，P 78次/分，R 20次/分，BP 139 mmHg/85 mmHg。发育正常，营养中等，左颈部肿胀，张口受限，局部皮肤发红、皮温高，扪及波动感。颈部CT：考虑左颈部，咽-喉咽左侧软组织感染性病变。胸部CT示（图 1B1）：胸骨后间隙见软组织肿胀及积气，考虑感染脓肿形成。血常规：WBC：19.25×10^9^/L，NEUT%：90%，RBC：3.8×10^12^/L，HGB：97.0 g/L；PLT：376×10^9^/L。诊疗经过：入院后急诊行颈部多间隙脓肿切开+纵隔持续负压引流术；创面分泌物培养：肺炎克雷伯菌感染，根据药敏选用抗生素治疗。患者病情平稳，体温正常，复查胸部CT（图 1B2）：胸骨后方软组织肿胀消退，可见引流管；于2017.12.23拔除引流管后出院。

## 3 讨论

急性纵隔感染大多数原因为食管穿孔（后纵隔）或经胸骨心脏手术后（前纵隔）或穿透伤。有时牙源性或扁桃体周围的感染可引起口咽部脓肿或严重的颈前间隙感染，脓液沿颈膜间隙扩散到纵隔内而导致急性纵隔感染^[3, 4]^；也可来源于口咽部的医源性穿孔、颈部创伤、会厌炎、腮腺炎、鼻窦炎、胸锁关节感染和非法静脉给药。此外颈部的任何操作，包括颈部淋巴结活检、甲状腺手术、气管切开术和纵隔镜检查等均可能造成纵隔感染，但比较罕见；因为颈部颈膜间隙与纵隔颈膜间隙相连接，感染可在重力、呼吸和胸腔内负压等作用下沿气管后间隙、气管前和颈动脉鞘间隙直接向下扩散，从而导致感染在胸廓内播散^[5]^；纵隔感染的主要临床表现为：通常急性起病，出现发热、白细胞增多、胸痛、吞咽困难及呼吸窘迫等症状。伴有颈部感染则易于识别，患者可主诉水肿、颈部疼痛、吞咽困难及吞咽痛^[6]^；根据美国疾病控制和预防中心制定的纵隔感染定义^[7]^，纵隔感染的诊断至少需符合以下标准中的一项：①离体纵隔组织或渗液中培养出微生物；②术中所见纵隔感染的证据；③有以下情况之一：胸痛、胸骨不稳定或发热（体温大于38 ℃）并同时伴有纵隔脓性分泌物或微生物血培养阳性或纵隔引流液中培养出微生物；急性纵隔感染死亡较高，尽管有广谱的抗生素治疗及CT成像，患者的死亡率仍高达25%-50%^[2]^；延误诊断、延误或不适当的纵隔引流是高死亡率的主要原因。从出现原发感染到住院治疗期间延误的时间从5天-22天不等，是导致患者死亡的一个重要因素^[1]^；随着感染沿颈深间隙扩散到纵隔，可出现颈部广泛的蜂窝组织炎、坏死、脓肿形成及脓毒血症^[8]^。并发症包括成人呼吸窘迫综合征、急性肾衰竭、肺炎等。在治疗上早期切开引流是治疗急性纵隔感染的金标准，但手术路径尤其是纵隔引流的最佳方式存在争议；为达到纵隔的最佳引流，文献中提到了多种路径，如剑突下路径，蛤壳式切口和胸骨正中切口，以及一些学者提出小切口的胸腔镜引流或清创术^[9, 10]^；我院陈成等还采用纵隔镜辅助行纵隔脓肿清创引流手术；在Wheatley等的研究^[11]^中，43例患者中的12例仅行颈部切开引流，20例最初行颈部引流的患者因感染扩散至下纵隔，随后行开胸引流术；我们收集17例患者均为口咽部感染继发前纵隔急性感染患者，我们认为有颌面外科的医生参与手术是必要的，可充分引流颌面部感染并防止感染继续蔓延至胸骨后间隙；以往纵隔感染的传统引流方法是切开引流放置引流条或引流管，患者需半卧位，使脓液或炎性渗出物在重力及咳嗽时挤压纵隔作用下被动流出体外，然后根据引流及敷料渗出情况，决定每日更换敷料及引流条的频率，必要时用双氧水、抗生素、生理盐水等反复冲洗脓腔，甚至需持续纵隔冲洗。尤其纵隔脓腔位置深，换药时疼痛明显，患者较为痛苦、换药时间长、工作量大，且每次换药者能否严格无菌操作，甚至会导致切口污染或二次感染。此外，引流管无法与引流腔充分接触，液体外流动力有限，易导致渗出液或脓液的积聚。

我们采用持续负压引流行前纵隔引流，其优点在于利用持续不间断负压吸引，在软组织中产生低于大气压的负压，使不断产生的作为细菌繁殖培养基的渗出物、脓液等有害物质及时持续地被清除^[12]^。本组病例均在胸骨后间隙行上下贯通双管负压引流术，放置引流管后缝合封闭切口，术后均未采取纵隔冲洗；所以切口不用广泛切开，减轻了对患者的损伤，减小了手术瘢痕，且两根引流管在必要时还可作对口引流，达到充分彻底引流之目的。

当然负压封闭引流术后，需要检查负压引流系统负压情况，保持引流系统密闭；我们使用一次性硅胶负压引流球，利用球囊是否回弹判断负压情况，球囊与引流管连接处为单向活瓣，可预防细菌逆行感染；该引流管不用冲洗创口，不用更换敷料及引流管，换药时间短、可以明显减轻患者的痛苦，也减轻了医生的工作量。利用负压引流球行负压引流，患者携带方便，可早期下地活动，在减轻患者创伤的同时，也减少了患者的心理负担^[13]^，更有利于患者的康复，防止长时间卧床引起的并发症。

本组患者治疗效果总体良好，虽有1例患者经治疗签字出院，但患者纵隔感染时间长，切开引流较晚，已经出现严重感染性休克，亦算作死亡病例；治疗严重急性前纵隔感染传统的胸骨切开引流，患者创伤大、心理难以承受，且医生需承担频繁更换敷料的任务；持续负压引流术改变了传统的脓肿切开引流治疗方式，减轻患者痛苦、能够充分引流纵隔积液，并且避免了敷料反复渗出而需要的换药；我们认为持续负压引流是治疗严重急性前纵隔感染的一种有效的方法。但该方法不能将引流管放置到中、后纵隔，因此对中、后纵隔感染引流存在局限性，是否考虑采用电视辅助胸腔镜手术（video-assisted thoracic surgery, VATS）、纵隔镜等辅助治疗，有待进一步优化。
